# Fabrication of 3-nm-thick Si_3_N_4_ membranes for solid-state nanopores using the poly-Si sacrificial layer process

**DOI:** 10.1038/srep14656

**Published:** 2015-10-01

**Authors:** Itaru Yanagi, Takeshi Ishida, Koji Fujisaki, Ken-ichi Takeda

**Affiliations:** 1 Hitachi Ltd., Central Research Laboratory, 1-280 Higashi-koigakubo, Kokubunji, Tokyo, 185-8603.

## Abstract

To improve the spatial resolution of solid-state nanopores, thinning the membrane is a very important issue. The most commonly used membrane material for solid-state nanopores is silicon nitride (Si_3_N_4_). However, until now, stable wafer-scale fabrication of Si_3_N_4_ membranes with a thickness of less than 5 nm has not been reported, although a further reduction in thickness is desired to improve spatial resolution. In the present study, to fabricate thinner Si_3_N_4_ membranes with a thickness of less than 5 nm in a wafer, a new fabrication process that employs a polycrystalline-Si (poly-Si) sacrificial layer was developed. This process enables the stable fabrication of Si_3_N_4_ membranes with thicknesses of 3 nm. Nanopores were fabricated in the membrane using a transmission electron microscope (TEM) beam. Based on the relationship between the ionic current through the nanopores and their diameter, the effective thickness of the nanopores was estimated to range from 0.6 to 2.2 nm. Moreover, DNA translocation through the nanopores was observed.

DNA sequencing with nanopores (i.e., nanopore sequencing) is a promising approach for achieving long-read, label-free, single-molecule DNA sequencing with very high throughput at low cost^1^,^2^,^3^,^4^,^5^. It is expected that personalized medicine will be provided in the future^6^. Therefore, both biological^7^,^8^,^9^,^10^,^11^ and solid-state^12^,^13^,^14^,^15^,^16^,^17^,^18^,^19^,^20^,^21^,^22^,^23^,^24^,^25^,^26^,^27^,^28^,^29^,^30^,^31^,^32^,^33^,^34^,^35^,^36^,^37^,^38^,^39^ nanopores have been intensively studied in recent years.

Compared with biological nanopores, solid-state nanopores have advantages in terms of robustness and possible large-scale integration. However, DNA sequencing with solid-state nanopores has not been demonstrated yet, although several ideas have been studied to achieve DNA sequencing based on solid-state nanopores^40^,^41^,^42^,^43^,^44^. The most famous DNA sequencing idea, common to both biological and solid-state nanopores, consists of detecting changes in the ionic current through a nanopore during the translocation of DNA and identifying the four types of nucleotides through these changes. However, many issues must be resolved to realize this idea with solid-state nanopores. In particular, from the standpoint of device fabrication, it is a significant challenge to stably fabricate ultrathin membranes and stably form nanopores in such thin membranes.

The optimal nanopore diameter for DNA sequencing has not yet been determined. However, the diameter of the nanopore should be small enough to prevent situations in which folded DNA enters into the nanopore or multiple DNA molecules enter into the nanopore simultaneously. For this reason, the diameter of the nanopore needs to be less than approximately 3 to 4 nm if nanopore sequencing is assumed to be performed with single-stranded DNA (ssDNA), the diameter of which is approximately 1.4 nm.

The spatial resolution of a nanopore sensor is determined by the thickness of the membrane and the size of the access resistance region^45^ around the nanopore. The distance between neighboring nucleotides in DNA is very short (approximately 0.4 nm). Consequently, thinning the membrane is a very important issue for highly accurate discrimination of each nucleotide in DNA.

Regarding nanopore fabrication, several techniques have been developed since the first demonstration of the fabrication of nanopores by ion-beam sculpting^22^. Currently, focused-electron beam etching using a transmission electron microscope (TEM) is the method most widely used to fabricate small-diameter nanopores^18^,^19^,^27^. In addition, nanopores have been fabricated by utilizing a helium ion microscope^23^,^24^,^25^, and dielectric breakdown of membranes^17^,^20^,^21^ was developed for high-throughput nanopore fabrication. These techniques enable fabrication of nanopores with diameters of less than 3 to 4 nm. Challenges remaining for the future include fabricating nanopores with low size variation in ultrathin membranes and reducing damage to the thin membranes during nanopore fabrication.

Regarding the formation of ultrathin membranes, two-dimensional materials have attracted attention. For example, graphene^30^,^31^,^32^,^33^,^34^,^35^,^36^, molybdenum disulfide^37^,^38^, and boron nitride^39^ have been considered and studied. Although these atomically thin materials are quite attractive membrane materials, stable mass production and control over their surface conditions remain issues. Another approach is to thin a membrane with semiconductor-related materials such as Si_3_N_4_ and hafnium oxide (HfO_2_). Recently, Larkin *et al.* reported the fabrication of 3- to 8-nm-thick HfO_2_ membranes using atomic layer deposition^14^. For Si_3_N_4_ membranes, thinning the membrane using reactive ion etching^12^,^13^ or helium ion beam etching^23^,^24^,^25^ has been demonstrated, and the thickness of the fabricated membranes is equal to or less than 5 nm. In addition, a method for transferring Si_3_N_4_ membranes to a quartz substrate (fishing method) has been proposed to fabricate 5-nm-thick Si_3_N_4_ membranes^16^.

Si_3_N_4_ is a traditional semiconductor-related material, and it is highly compatible with semiconductor processes. Therefore, it is highly desirable to use Si_3_N_4_ as a membrane material for solid-state nanopores. However, until now, stable wafer-scale fabrication of Si_3_N_4_ membranes with thicknesses less than 5 nm has not been reported, although a further reduction in thickness is desired. In this study, to fabricate thinner Si_3_N_4_ membranes with thicknesses of less than 5 nm in a wafer, a new fabrication process that employs a polycrystalline-Si (poly-Si) sacrificial layer was proposed and evaluated. This fabrication process significantly minimizes damage to the membrane. Using this process, Si_3_N_4_ membranes with thicknesses of 3 nm were stably fabricated with small thickness variation. After fabricating the membranes, nanopores were fabricated through focused-electron-beam etching using a TEM. The effective thicknesses of the fabricated nanopores were estimated based on the relationship between the ionic current through the nanopores and their diameter. Finally, long-term stability during measurement of DNA translocation through the nanopores and the characteristics of DNA translocation events were investigated.

## Results

### Membranes fabricated using the poly-Si sacrificial layer process

**Figure 1:**
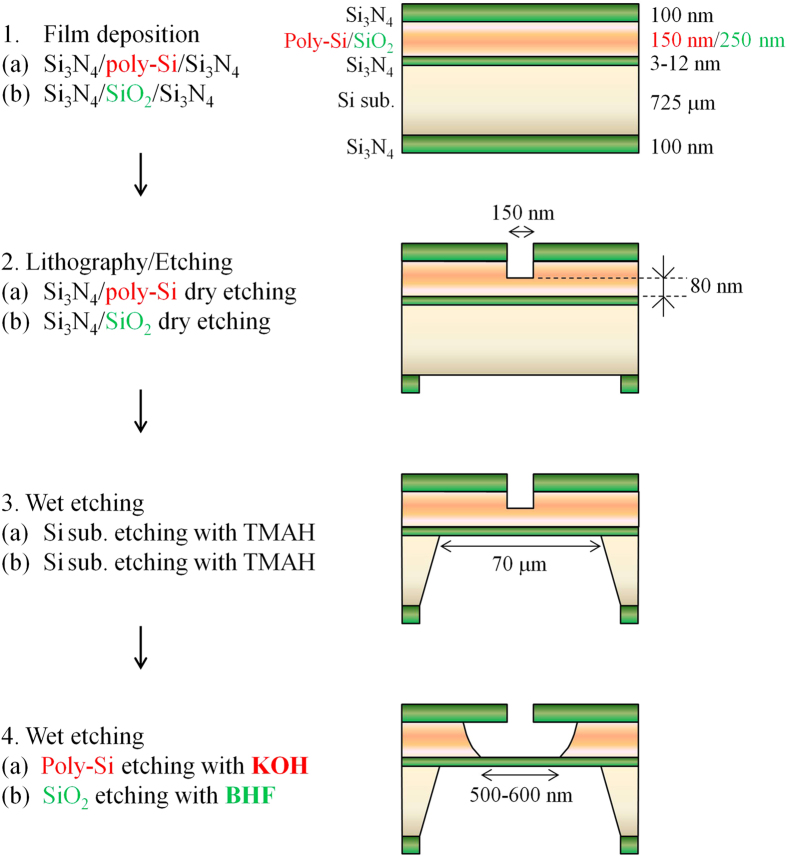
Process flow for membrane fabrication. (**a**) Process flow for the fabrication of membranes using the poly-Si sacrificial layer process. (**b**) Process flow for the fabrication of membranes using the SiO_2_ sacrificial layer process.

Si_3_N_4_ membranes were fabricated using 8-inch Si wafers. The process flow for fabricating the membranes is shown in Fig. 1. This figure depicts two fabrication processes. The first method is the poly-Si sacrificial layer process (a). The second method is the SiO_2_ sacrificial layer process (b), which was employed in our previous work and in other studies^17^,^26^. These processes have the advantage of enabling the fabrication of membranes with a small area of approximately 500 nmϕ, which can reduce the probability of initial breakage of the membrane. The most important difference between these two processes is the different etchants used to remove the sacrificial layer. The Si_3_N_4_ layer was formed via low-pressure chemical vapor deposition (LPCVD), and potassium hydroxide (KOH) aqueous solution will not etch this layer because of its strong chemical resistance against KOH aqueous solution^46^,^47^. However, buffered hydrofluoric acid (BHF) aqueous solution can etch the Si_3_N_4_ layer, although its etching rate is slow^46^,^47^. Therefore, the poly-Si sacrificial layer process has the potential to stably fabricate thinner Si_3_N_4_ membranes.

**Figure 2:**
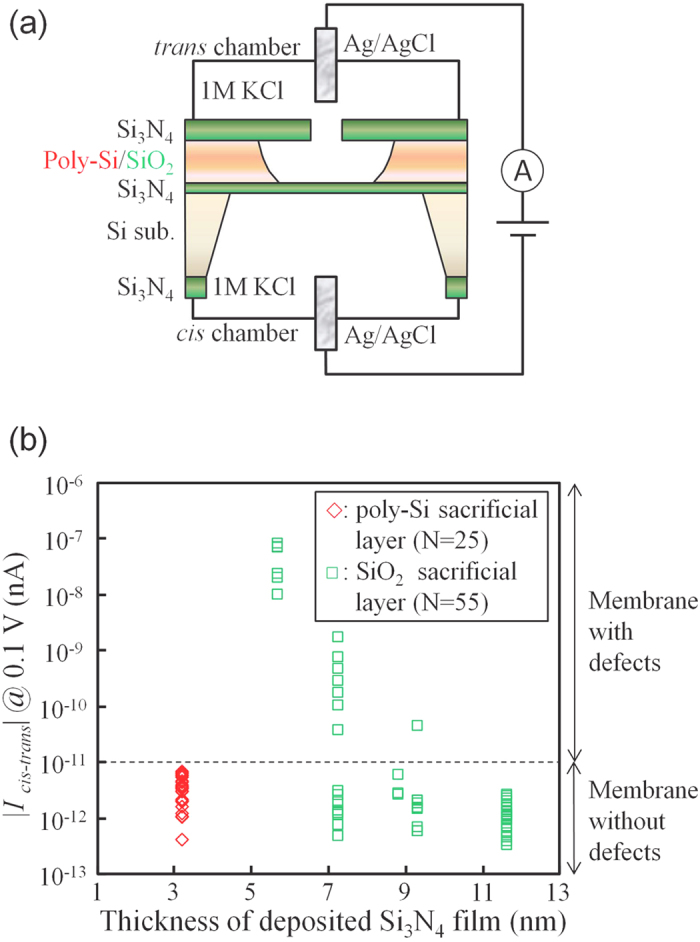
Measurement of leakage current through the fabricated membranes. (**a**) Setup for the measurement of leakage current through the membranes. (**b**) Dependence of the leakage current on the thickness of the deposited Si_3_N_4_ film at 0.1 V. Green symbols represent the leakage current through membranes fabricated using the SiO_2_ sacrificial layer process (55 different membranes were measured). Red symbols represent the leakage current through membranes fabricated using the poly-Si sacrificial layer process (25 different membranes were measured).

**Figure 3:**
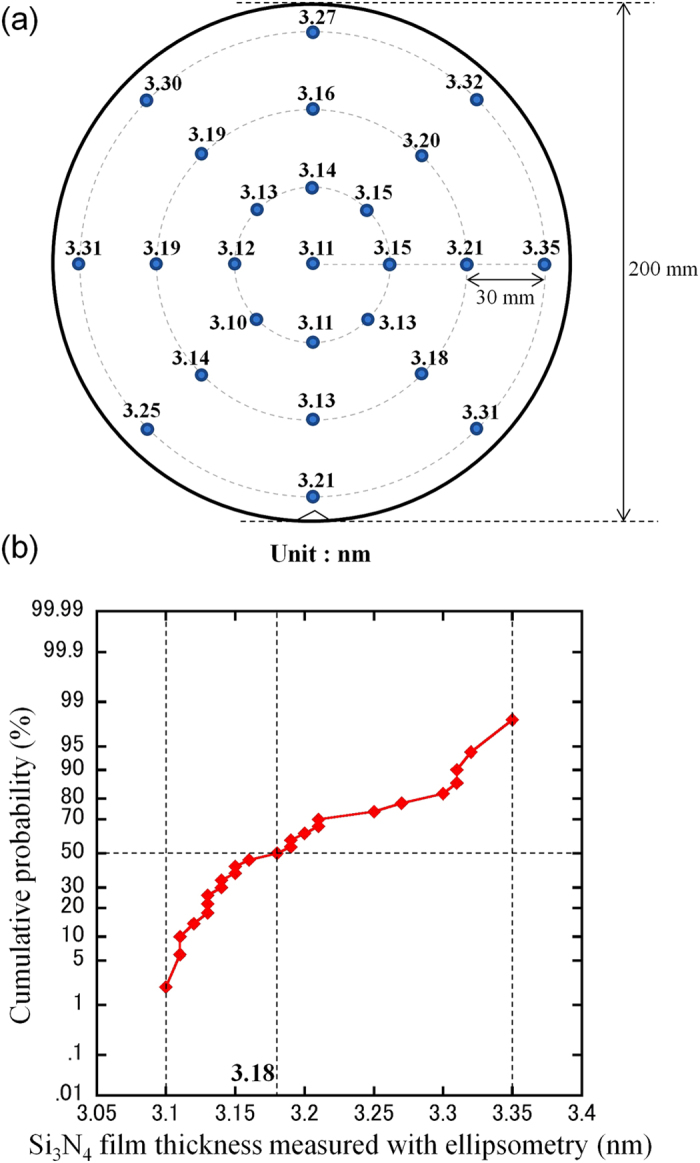
Thickness of Si3N4 films measured using ellipsometry. (**a**) Thickness measured at each point on the wafer (25 points). The reflective index was set to 2.0. (**b**) Cumulative probability of the measured thickness values.

To determine whether the fabricated membranes contain initial defects or breakage, the ionic leakage current through the membranes was measured. Figure 2 shows the leakage current through the membranes fabricated using the above two processes. The setup for the measurement is shown in Fig. 2a. Two chambers (*cis* and *trans* chambers) were separated by the Si_3_N_4_ membrane. Both chambers were filled with a 1 M KCl aqueous solution. Ag/AgCl electrodes were immersed in the aqueous solutions and connected to a voltage source and an ammeter. The voltage applied was 0.1 V. Figure 2b shows the dependence of the leakage current (*I*_*cis-trans*_) through the membrane on the thickness of the deposited bottom Si_3_N_4_ film. The green symbols represent the leakage current through the membranes fabricated using the SiO_2_ sacrificial layer process (55 different membranes were measured), and the red symbols represent the leakage current through the membranes fabricated using the poly-Si sacrificial layer process (25 different membranes were measured). Each *I*_*cis-trans*_ was measured 1 second after the voltage was applied. The threshold current used to determine whether a membrane contained initial defects or breakage was determined to be 10 pA. The thickness of each deposited Si_3_N_4_ film was defined as the average thickness of 25 points on each wafer, which was measured by ellipsometry using a refractive index of 2.0 (the locations of the measurement points are shown in Fig. 3a). The measurement of these thicknesses was performed immediately after the deposition of the bottom Si_3_N_4_ layer and before the deposition of the poly-Si or SiO_2_ layer. As shown in Fig. 2a, for the membranes fabricated using the SiO_2_ sacrificial layer process, high leakage current was observed when the thickness of the deposited Si_3_N_4_ film was less than or equal to 7 to 9 nm. This result indicates that the SiO_2_ sacrificial layer process cannot fabricate membranes with thicknesses of less than 7 nm. In contrast, the membranes fabricated using the poly-Si sacrificial layer process exhibited no significant leakage current, even when the thickness of the deposited Si_3_N_4_ film was 3.18 nm.

**Figure 4:**
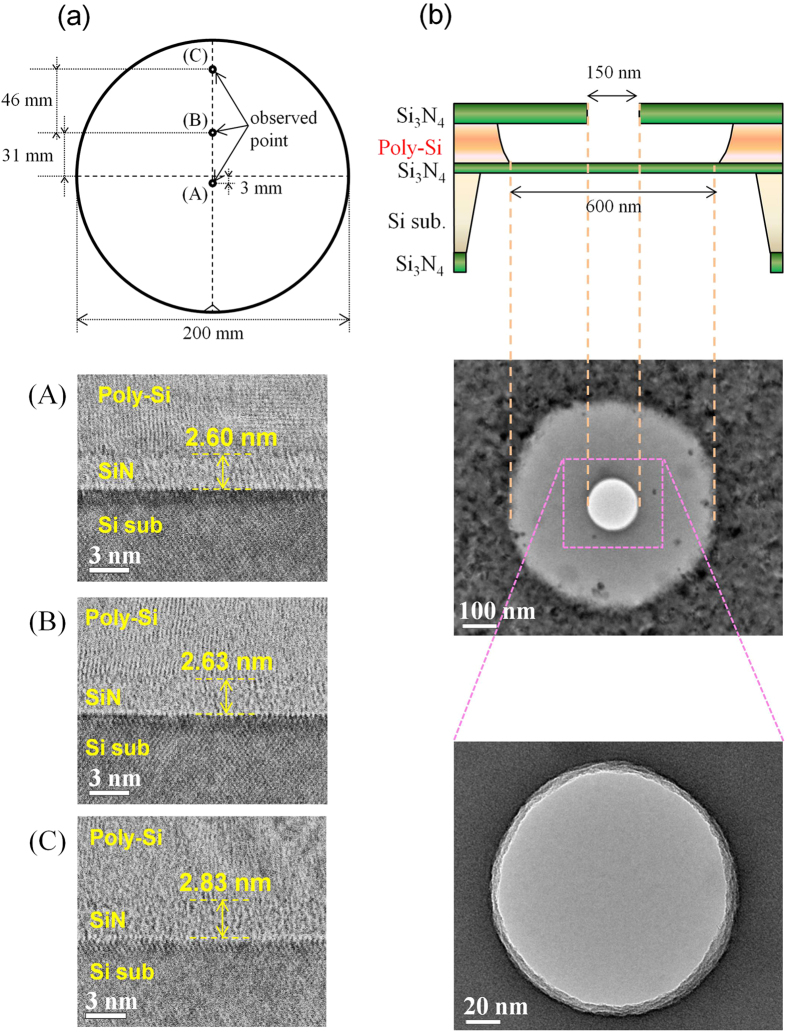
Cross-sectional STEM images of the Si_3_N_4_ layer and top views of the Si_3_N_4_ membrane. (**a**) The Si_3_N_4_ layer at three different points on the wafer was observed at 2000 k-fold magnification. (**b**) A TEM image of the entire membrane at 20 k-fold magnification. A magnified TEM image at 100 k-fold magnification.

The remainder of the study focused on evaluating these 3.18-nm-thick membranes. Figure 3 presents detailed information on the thickness of the deposited Si_3_N_4_ films measured using ellipsometry. The film thickness at each point on the wafer is shown in Fig. 3a, and the cumulative probability of the thicknesses is shown in Fig. 3b. The variation in the film thickness was quite small (3.10–3.35 nm), and the average thickness was 3.18 nm. Figure 4a presents cross-sectional scanning transmission electron microscope (STEM) images of the Si_3_N_4_ films at three points on the wafer ((A), (B), and (C) in Fig. 4a). From these STEM images, the Si_3_N_4_ film thickness was found to be approximately 2.7 nm, which is in fairly good agreement with the thickness measured using ellipsometry. Top-view TEM images of the membrane are shown in Fig. 4b. This figure confirms that the poly-Si sacrificial layer can be removed by etching with KOH aqueous solution and that clean Si_3_N_4_ membranes can be fabricated. From the above results in Figs 3 and 4, it can be concluded that Si_3_N_4_ membranes with thicknesses of approximately 3 nm can be fabricated using the poly-Si sacrificial layer process.

### Electrical properties of membranes fabricated with nanopores

**Figure 5:**
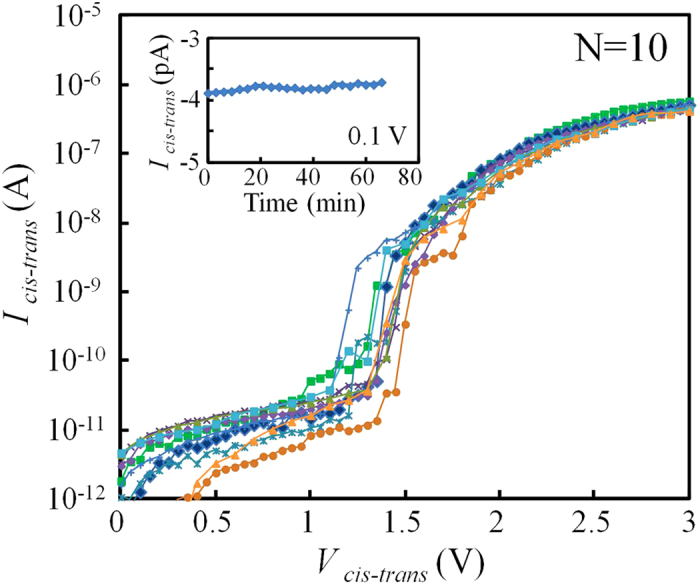
Current-voltage characteristics through Si_3_N_4_ membranes. Current-voltage characteristics of 10 different membranes were measured. The voltage was swept from 0 to 3 V in steps of 0.05 or 0.1 V. The inset figure shows a long-term continuous measurement of a membrane at 0.1 V.

The dielectric breakdown voltage of the fabricated membranes was investigated and is shown in Fig. 5. The setup for the measurements was the same as that shown in Fig. 2. Each *I*_*cis-trans*_ point was measured one second after each voltage was applied. Dielectric breakdown occurred when the applied voltage (*V*_*cis-trans*_) reached 1 to 1.5 V. This dielectric breakdown voltage is markedly lower than that of 10-nm-thick Si_3_N_4_ membranes, which is approximately 7 to 10 V^17^,^20^. The inset figure shows a long continuous measurement of *I*_*cis-trans*_ at 0.1 V. The change in *I*_*cis-trans*_ was negligibly small (approximately 0.1 pA), and dielectric breakdown of the membrane did not occur during a one-hour-long measurement. Therefore, the voltage used for ionic current measurements was usually set to 0.1 V.

**Figure 6:**
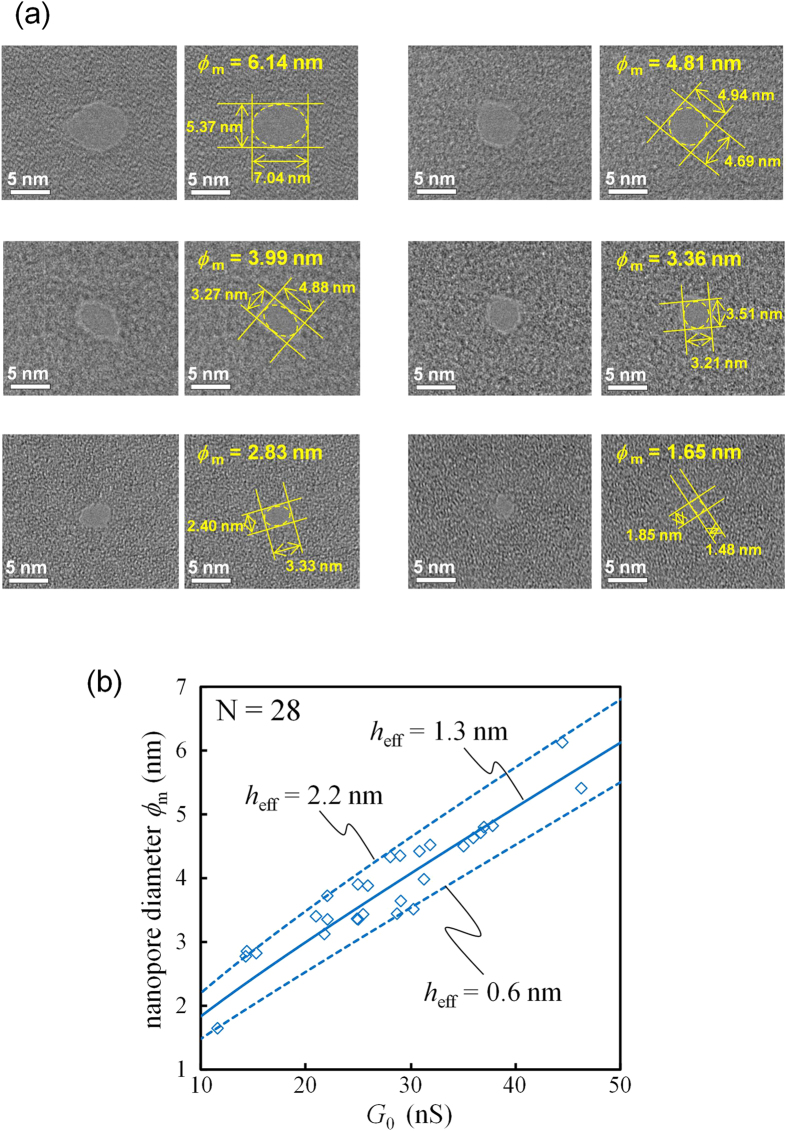
TEM images of nanopores and relationship between the ionic current through nanopores and their diameter. (**a**) TEM images of fabricated nanopores at 400 k-fold magnification. Each left image shows the raw image of each right image. (**b**) Relationship between ionic conductance through nanopores (*G*_0_) and their diameters (ϕ_m_). A total of 28 points are plotted within ϕ_m_ = 1.65 to 6.14 nm.

Figure 6a presents TEM images of nanopores fabricated via focused-electron beam etching using a TEM. Nanopores with diameters of approximately 2 to 6 nm were fabricated. The mean diameter (ϕ_m_) was defined with an ellipsoidal approximation as


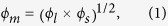


where ϕ_l_ and ϕ_s_ are the major and minor axes, respectively, of the nanopore measured from the TEM image. The relationship between ϕ_m_ and the conductance of the ionic current through the nanopore (*G*_0_) is illustrated in Fig. 6b. The currents were recorded 3 to 5 seconds after the voltage (0.1 V) was applied. The plotted measurements could be fitted with the theoretically calculated lines obtained as follows^13^,^45^:


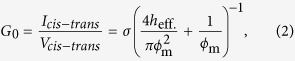


where *h*_eff_ is the effective height of the nanopore and σ = 0.104 S/cm is the measured conductance of the KCl buffer solution at 21.0 °C. The calculation with a *h*_eff_ of 1.3 nm is the central fitting line, and the variation in *h*_eff_ is within 0.6 to 2.2 nm. According to previous studies^12^,^13^,^14^,^16^,^17^, *h*_eff_ is smaller than the actual membrane thickness. Our result is also consistent with this trend. The *I-V* characteristics of the 150-nmϕ opening window in the top Si_3_N_4_ layer are shown in Supplementary Section SI-1. The ionic conductance through the Si_3_N_4_ opening window is approximately 770–930 nS. This value is approximately one to two orders of magnitude higher than the ionic conductance through the nanopores. Therefore, the Si_3_N_4_ opening window does not supply significant series resistance to the system. The *I-V* characteristics of the nanopores are shown in Supplementary Section SI-2. Linear and symmetric *I-V* characteristics are confirmed.

**Figure 7:**
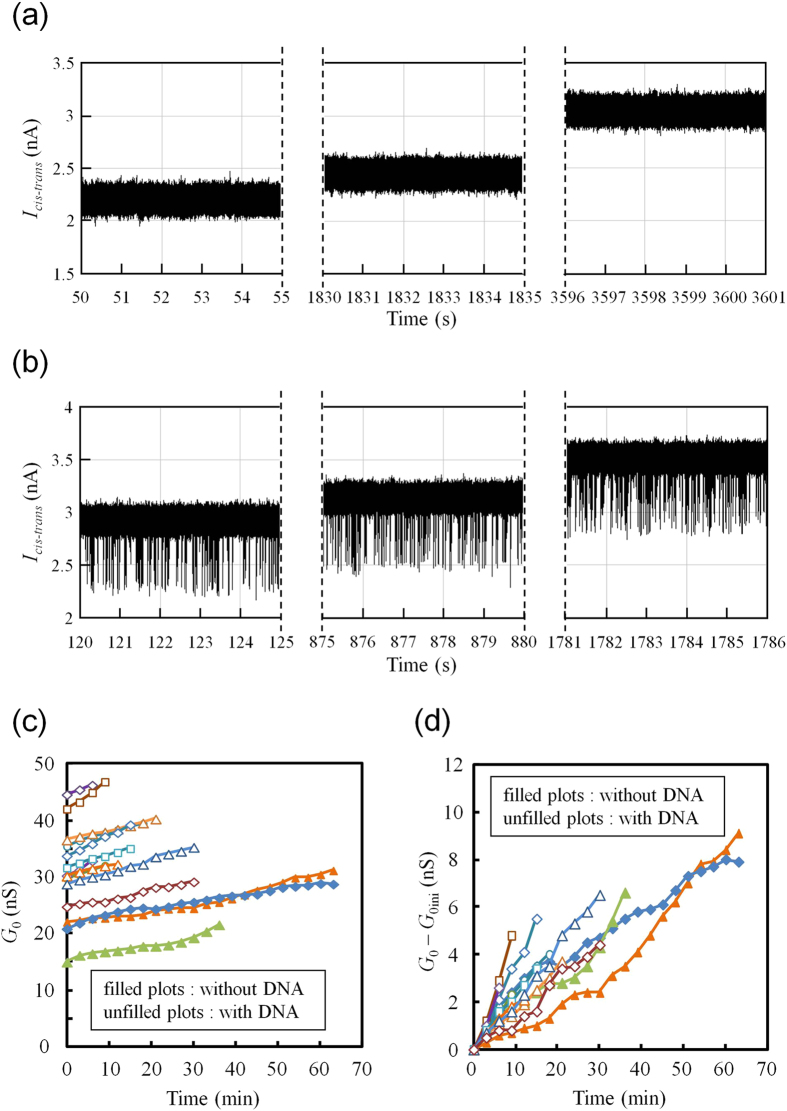
Long-term continuous measurement of current through nanopores. (**a**) Continuous measurement of current through a nanopore at 0.1 V for one hour. Both *cis* and *trans* chambers were filled with 1 M KCl buffer solution. dsDNA was not applied. (**b**) Continuous measurement of current through a nanopore at 0.1 V for half an hour. The *cis* chamber was filled with 1 M KCl buffer solution with 20 nM 1 kbps dsDNA. The *trans* chamber was filled with 1 M KCl buffer solution. (**c**) Changes in baseline conductance (*G*_0_) with time. (**d**) Changes in *G*_0_ from the initial baseline conductance (*G*_0ini_). The current was plotted every 3 minutes.

Long-term continuous measurements of *I*_*cis-trans*_ through the nanopores at 0.1 V are shown in Fig. 7. Figure 7a shows *I*_*cis-trans*_ thorough a nanopore with a ϕ_m_ of 3.36 nm without applying DNA into the *cis* chamber. Figure 7b shows *I*_*cis-trans*_ thorough a nanopore with a ϕ_m_ of 3.65 nm after adding 20 nM 1 kbps double-stranded DNA (dsDNA) into the *cis* chamber. Typical *I*_*cis-trans*_ noise power spectrums are shown in Supplementary Section SI-3. After adding dsDNA into the *cis* chamber, ionic current blockades were frequently observed, which indicated the occurrence of dsDNA translocation through the nanopores. However, the baseline *I*_*cis-trans*_ current increased over time, which indicated widening of the nanopores. TEM images of the nanopores before and after the measurement of *I*_*cis-trans*_ are shown in Supplementary Section SI-4. The widening of the nanopores was confirmed after the measurements. Such widening of nanopores after ionic-current measurements has been reported previously^27^. This increase in current was not observed prior to fabrication of the nanopores (inset of Fig. 5). Therefore, it is assumed that areas of the membrane near the edges of nanopores are weaker than the other areas. Figure 7c shows the change in baseline conductance (*G*_0_) with time, and Fig. 7d shows the change in *G*_0_ from the initial baseline current (*G*_0ini_) that was measured at the beginning of the measurement period. Unfilled/filled symbols represent the data obtained from measurements with/without dsDNA in the *cis* chamber. The increase in *G*_0_ was approximately 5 nS for a half hour at 0.1 V.

**Figure 8:**
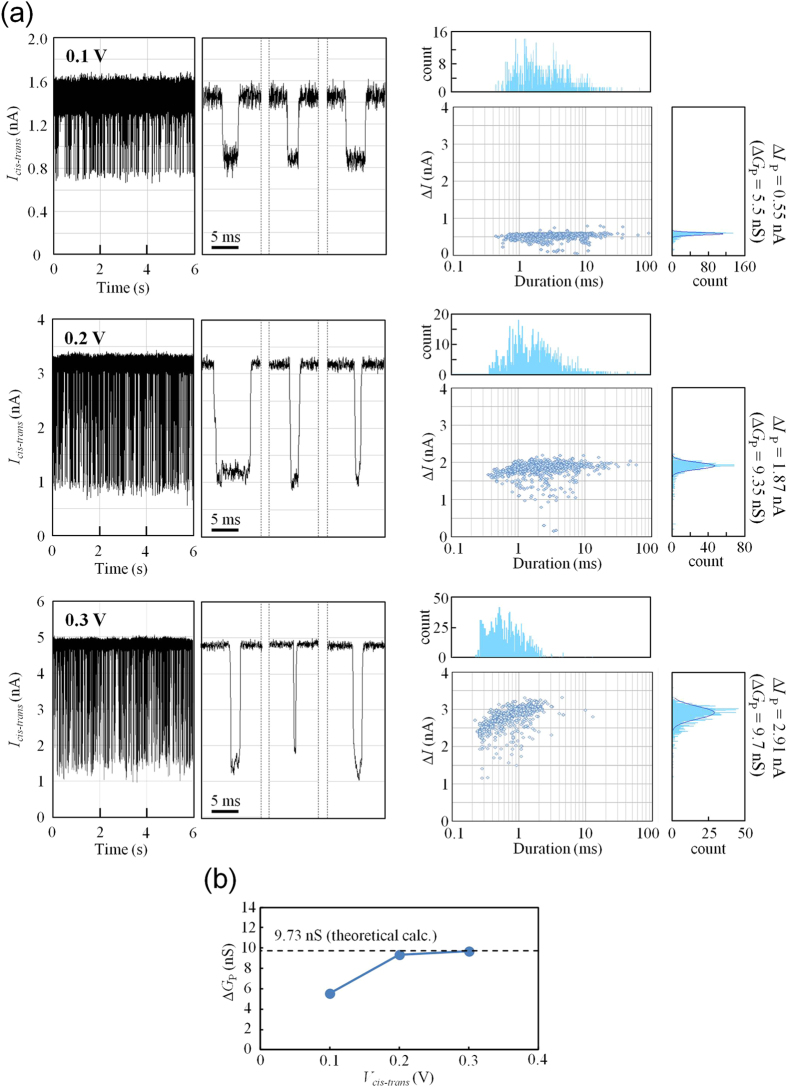
Voltage dependency shown in scatter plots and histograms of ionic-current blockades. (**a**) The left figures show time traces of ionic current through a nanopore with a ϕ_m_ of 2.88 nm. The voltage applied was 0.1 to 0.3 V. Magnified views show typical ionic-current-blockade events. The right figures show scatter plots and histograms of the current-blockade events at 0.1 to 0.3 V. (**b**) Dependence of the mean conductance blockade (Δ*G*_P_) on the applied voltage. The dashed line represents the theoretically predicted value of Δ*G* derived from equations (3–5).

Figure 8(a) shows scatter plots of the voltage dependency and histograms of the ionic-current blockades. This voltage dependency was measured using the same nanopore with a ϕ_m_ of 2.88 nm. It is reasonable that the duration of the ionic-current blockade became shorter as the applied voltage became higher because the speed of DNA translocation through the nanopores increased with increasing voltage. However, the depth of the mean ionic-current blockade (Δ*I*_P_) showed an anomalous increase with increasing voltage (Δ*I*_P_ was calculated from Gaussian fits to each histogram). Figure 8(b) shows the dependence of the mean conductance blockade (Δ*G*_P_ = Δ*I*_P_*/V*_*cis-trans*_) on the applied voltage. Δ*G*_P_ at a lower voltage (0.1 V) was significantly smaller than Δ*G*_P_ at higher voltages (0.2–0.3 V). In our experiments with other nanopores (see Supplementary Section SI-5), Δ*G*_P_ at 0.1 V was 3.3 nS (the minimum value we observed) to 7.0 nS (the maximum value we observed). These Δ*G*_P_ values at 0.1 V are markedly lower than Δ*G*_P_ derived from a theoretical prediction discussed later.

Similar phenomena (i.e., an anomalous increase in Δ*G*_P_ with increasing voltage) have been reported in several papers^23^,^25^,^28^,^29^. Recently, Carlsen *et al.* proposed a model to interpret these phenomena^25^. According to the model, positive counter ions surrounding dsDNA counteract the conductance blockade in the presence of low electrical fields, and these counterions are gradually removed from the dsDNA as the electrical field increases.

We theoretically estimated Δ*G*_P_ at high voltages when counterions do not surround dsDNA. In this study, the geometric model proposed in ref. 25 was employed. Δ*G* is expressed as follows,


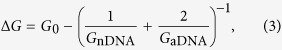



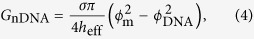



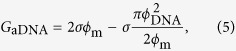


where *G*_nDNA_ is the conductance of the inside of the nanopore during DNA translocation, *G*_aDNA_ is the conductance of the access resistance region during DNA translocation, and ϕ_DNA_ is the diameter of dsDNA (2.2 nm). *h*_eff_ was assigned to be 1.3 nm. The estimated Δ*G* from equations (3)–(5) is 9.73 nS. This value is in good agreement with the experimental results derived from high voltages (0.2–0.3 V). The behavior wherein Δ*G* approaches the theoretically predicted value with increasing voltage is consistent with the results reported in ref. 25.

## Discussion

The poly-Si sacrificial layer process was proposed and demonstrated to fabricate thin Si_3_N_4_ membranes with thicknesses of less than 5 nm. The poly-Si sacrificial layer process enables the fabrication of membranes with thicknesses of approximately 3 nm, whereas the conventional SiO_2_ sacrificial layer process cannot stably fabricate membranes when the thickness of the deposited Si_3_N_4_ film is less than or equal to 7 to 9 nm. We believe that this difference primarily resulted from the different etchants used to remove the sacrificial layer. KOH aqueous solution cannot etch Si_3_N_4_ membranes, whereas BHF aqueous solution can etch Si_3_N_4_ membranes. However, the rate of etching of the Si_3_N_4_ layer with BHF aqueous solution is very slow, and it remains incompletely explained why the SiO_2_ sacrificial layer process could not stably fabricate membranes when the thickness of the deposited Si_3_N_4_ film was less than or equal to 7 to 9 nm. The measured etching rate of the Si_3_N_4_ layer was 0.17 nm/min in the presence of BHF (HF:NH_4_F = 1:60), whereas the total etching time for the SiO_2_ sacrificial layer was set to 8.5 min. Even when both sides of the Si_3_N_4_ membranes were exposed to the BHF aqueous solution during the etching process, the Si_3_N_4_ membranes could not be etched more than 3 nm, and a membrane with a deposited Si_3_N_4_ film 7 nm in thickness should have been fabricated stably. We believe that the result is attributable to the following two factors. The first factor is oxidation occurring on the surface of the bottom Si_3_N_4_ layer when the SiO_2_ sacrificial layer was deposited onto it. This process leads to weakening of the chemical resistance of the Si_3_N_4_ layer against BHF aqueous solution. The second factor is the total stress on the membrane. Compared with membranes fabricated using the poly-Si sacrificial layer process, membranes fabricated using the SiO_2_ sacrificial layer process had higher tensile stresses. In this study, Si_3_N_4_ with a tensile stress of approximately 900 MPa, SiO_2_ with a tensile stress of approximately 150 MPa, and poly-Si with a compressive stress of approximately 250 MPa were used. Therefore, the total stress of the membranes fabricated using the SiO_2_ sacrificial layer process was supposed to be a tensile stress of approximately 360 MPa, which was higher than that of the membranes fabricated using the poly-Si sacrificial layer process (approximate tensile stress of 210 MPa). Higher tensile stresses of a membrane may lead to a decrease in its mechanical stability.

The mean effective thickness of nanopores fabricated in membranes with thicknesses of 3 nm was found to be approximately 1.3 nm. According to the results reported by Lee *et al.*^16^, the effective thickness of nanopores in 5-nm-thick Si_3_N_4_ membranes is approximately 2.4 nm. Therefore, thinning of the effective thickness is also confirmed to be associated with thinning of the physical thickness of the membrane.

According to long-term continuous measurements of the current through nanopores at 0.1 V, the current increased over time. This result was caused by widening of the nanopores. Considering that there was no increase in the current for one hour during the measurement of the current through membranes without nanopores, it is assumed that the parts of the membrane near the edges of nanopores are weaker than the other parts. It is assumed that one of the possible causes of this degradation is irradiation by the TEM beam. We think that the conditions and parameters of our TEM beam etching have not yet been optimized. In ref. 27, van den Hout *et al.* reported that the widening of nanopores during ionic-current measurements could be mitigated by optimizing the TEM beam size and other parameters. We also need to optimize the parameters of TEM beam etching for such thin membranes based on that report^27^. In addition, it is also important to fabricate more robust Si_3_N_4_ membranes by improving the film formation process. To obtain more robust membranes, the chemical composition should be more stoichiometric than the current composition.

Conductance blockades (Δ*G*) during DNA translocation events increased as the voltage increased. Δ*G* at low voltages (0.1 V) was less than that at high voltages (0.2–0.3 V), and Δ*G* at high voltages was in good agreement with the theoretical prediction (approximately 9.7 nS). This behavior is consistent with that previously reported by Carlsen *et al.*^25^. However, our observed Δ*G* at high voltages is not the highest value among those previously reported for Si_3_N_4_ nanopores. In ref. 13, a Δ*G* of approximately 13 nS was reported using an 8-nm-thick Si_3_N_4_ membrane and 3 kbps dsDNA. We cannot explain why such a high Δ*G* was observed using a thicker membrane than ours, and we think that a more extended model is required to explain any DNA translocation events.

In conclusion, we fabricated Si_3_N_4_ membranes with thicknesses of approximately 3 nm using the poly-Si sacrificial layer process. The mean effective thickness of the nanopores fabricated in the membrane was approximately 1.3 nm. This ultrathin membrane could be fabricated across a wafer with extremely low variation in thickness, and we thus conclude that the poly-Si sacrificial layer process is a promising approach for fabricating ultrathin membranes with solid-state nanopores. We believe that the thickness of 3 nm is not a limit; by forming more stoichiometric Si_3_N_4_ films and improving the nanopore fabrication method, sub-3-nm-thick Si_3_N_4_ membranes with nanopores could be fabricated.

## Methods

### Fabrication of membranes

The membranes were fabricated on an 8-inch silicon wafer with a thickness of 725 μm. First, a Si_3_N_4_ layer with a thickness of 3 to 12 nm was deposited using low-pressure chemical vapor deposition (reacting gases: SiH_4_-NH_3,_ 650 °C for 4 min), followed by measurement of the thickness of the Si_3_N_4_ layer with a single-wavelength ellipsometer (wavelength: 632.8 nm; MARY-102SM, Five Lab Co., Ltd., Japan). After the measurement, a multilayer of SiO_2_/Si_3_N_4_ (250/100 nm) or poly-Si/Si_3_N_4_ (150/100 nm) was deposited onto the front of the wafer, and a Si_3_N_4_ layer with a thickness of 100 nm was deposited onto the backside of the wafer. The top Si_3_N_4_ layer was etched in circular areas with a diameter of 150 nm by reactive-ion etching, as was the backside Si_3_N_4_ layer in corresponding 1038 × 1038-μm^2^ square areas, followed by etching of the silicon substrate with tetramethylammonium hydroxide (TMAH) at 85 °C for 9 hours. During etching of the silicon substrate, the front surface of the wafer was covered with protective film (ProTEK®B3 primer and ProTEK®B3, Brewer Science, Inc.). The protective film was removed by acetone after etching of the silicon substrate. Finally, the SiO_2_ or poly-Si layer in each circular area was etched with buffered hydrofluoric acid (BHF: HF:NH_4_F = 1:60 for 8.5 min) or potassium hydroxide (28 wt% solution of KOH for 16 min) at room temperature, and thin Si_3_N_4_ membrane portions with thicknesses of 3 to 12 nm were fabricated.

### Observation and fabrication of nanopores by TEM

Cross-sectional images of the Si_3_N_4_ layers were obtained using a scanning transmission electron microscope (HD 2700, 200 kV, Hitachi High-Technologies Corp.). Observations of the top of the Si_3_N_4_ membranes and nanopore fabrication were performed using a field-emission transmission electron microscope (JEM-2100F (HRP), 200 kV, JEOL, Ltd.). The electron flux used to fabricate the nanopores was approximately 1 × 10^8^ to 1 × 10^9^ e^−^ nm^−2^ s^−1^, and the irradiation time was approximately 5 seconds.

### Setup for measurement of current through nanopores

Initially, the membrane was mounted onto a custom-built acrylic flow cell. The flow cell has two chambers (each with a volume of 90 μL) separated by the membrane. One is a *cis* chamber and the other is a *trans* chamber. For measurements without DNA, both chambers were filled with buffer solution (1 M potassium chloride (KCl), 10 mM Tris-HCl, and 1 mM EDTA buffer at pH 7.5). For measurements with DNA, the *cis* chamber was filled with 1 M KCl buffer solution with 20 nM 1 kbps dsDNA (NoLimits, Fermentas, Burlington, Ontario, Canada). To ensure electrical contact with each aqueous solution, an Ag/AgCl electrode was immersed into each aqueous solution.

The measurements of ionic current shown in Figs 2 and 5 were performed using a 4156B Precision Semiconductor Parameter Analyzer (Agilent Technologies, Inc.). The current was measured one second after the voltage was applied. The measurements of ionic current shown in Figs 6, 7, 8 and the inset of Fig. 5 were performed using a patch-clamp amplifier (Axopatch 200B, Axon Instruments, Union City, CA). The detected current was low-pass-filtered with a cut-off frequency of 10 kHz using a four-pole Bessel filter and then digitized with an NI USB-6281 18-bit DAQ AD converter (National Instruments, Austin, TX) at 50 kHz. Finally, the current was recorded on the hard disk of a personal computer. These procedures and measurements were performed at room temperature. Event analysis of ionic-current blockades was performed using the OpenNanopore software (École polytechnique fédérale de Lausanne).

## Additional Information

**How to cite this article**: Yanagi, I. *et al.* Fabrication of 3-nm-thick Si_3_N_4_ membranes for solid-state nanopores using the poly-Si sacrificial layer process. *Sci. Rep.* **5**, 14656; doi: 10.1038/srep14656 (2015).

## Supplementary Material

Supplementary Information
