# Supplementary material for: Fabrication of 3-nm-thick Si3N4 membranes for solid-state nanopores using the poly-Si sacrificial layer process
Source: Sci Rep. 2015 Oct 1;5:14656. doi: 10.1038/srep14656 (PMC4589763; doi:10.1038/srep14656)
Supplement: Supplementary Information [file srep14656-s1.pdf]

Supplementary Information for
Fabrication of 3-nm-thick Si_3N_4 membranes for solid-state nanopores using the poly-Si sacrificial layer process

Itaru Yanagi*, Takeshi Ishida, Koji Fujisaki and Ken-ichi Takeda

Hitachi Ltd., Central Research Laboratory, 1-280 Higashi-Koigakubo, Kokubunji, Tokyo, 185-8603

The Supplementary Information includes:

SI-1. *I-V* characteristics of Si_3N_4 opening windows

SI-2. *I-V* characteristics of nanopores

SI-3. Noise spectrums in measurements of the ionic current through nanopores

SI-4. TEM images of nanopores before and after ionic-current measurements

SI-5. Ionic-current blockades with several nanopores at 0.1 V

SI-1. *I-V* characteristics of Si_3N_4 opening windows

To measure the *I-V* characteristics of the opening window in the top Si_3N_4 layer, the bottom Si_3N_4 membrane was removed with hydrofluoric acid (HF:water = 1:100 for 10 min). The etching rate of Si_3N_4 was 0.2 - 0.3 nm/min. Therefore, the top Si_3N_4 layer was also slightly etched by 4-6 nm. As a result, the thickness of the top Si_3N_4 layer decreased from 100 nm to 94-96 nm (Fig. S-1a). Fig. S-1b shows the *I-V* characteristics of the top Si_3N_4 opening window obtained using 1M KCl. Six different chips were measured. The conductance of the Si_3N_4 opening windows was 770 – 930 nS.

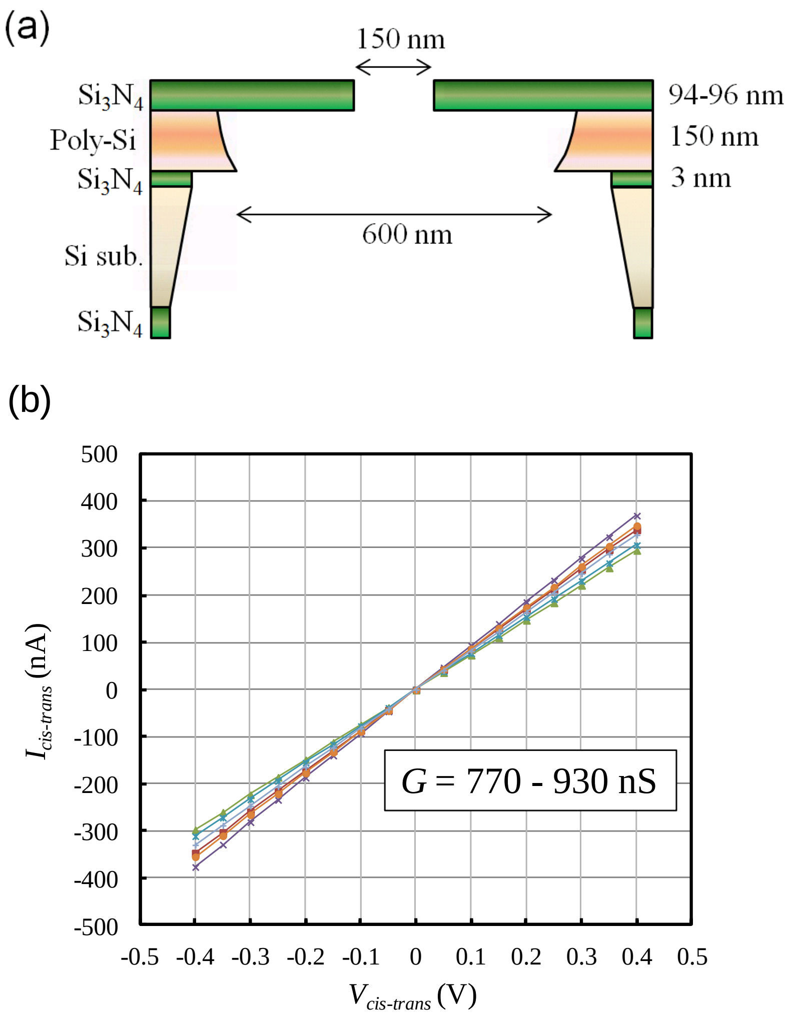

Fig. S-1

SI-2. *I-V* characteristics of nanopores

Fig. S-2a and Fig. S-2b show the *I-V* characteristics of 8 different nanopores in 1 M KCl. Fig. S-2b is a magnified view of Fig. S-2a with a voltage range of -0.1 to 0.1 V. The current was recorded 3 to 5 seconds after the voltage was applied.

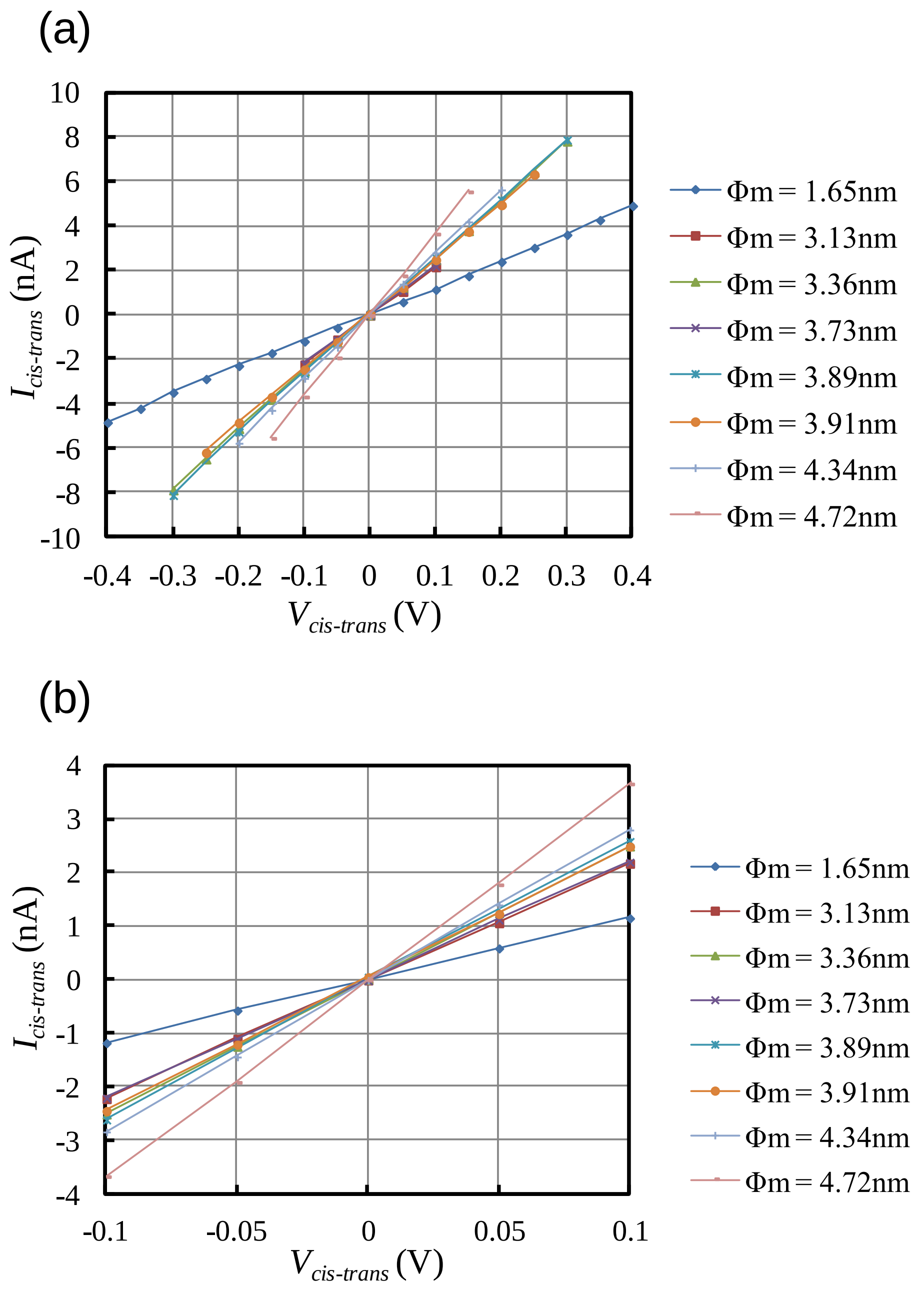

Fig. S-2

SI-3. Noise spectrums in measurements of the ionic current through nanopores

Noise spectrums of 3 different nanopores are shown in Fig. S-3. The ionic current was measured at 0.1 V in 1 M KCl. The detected current was low-pass-filtered with a cut-off frequency of 10 kHz.

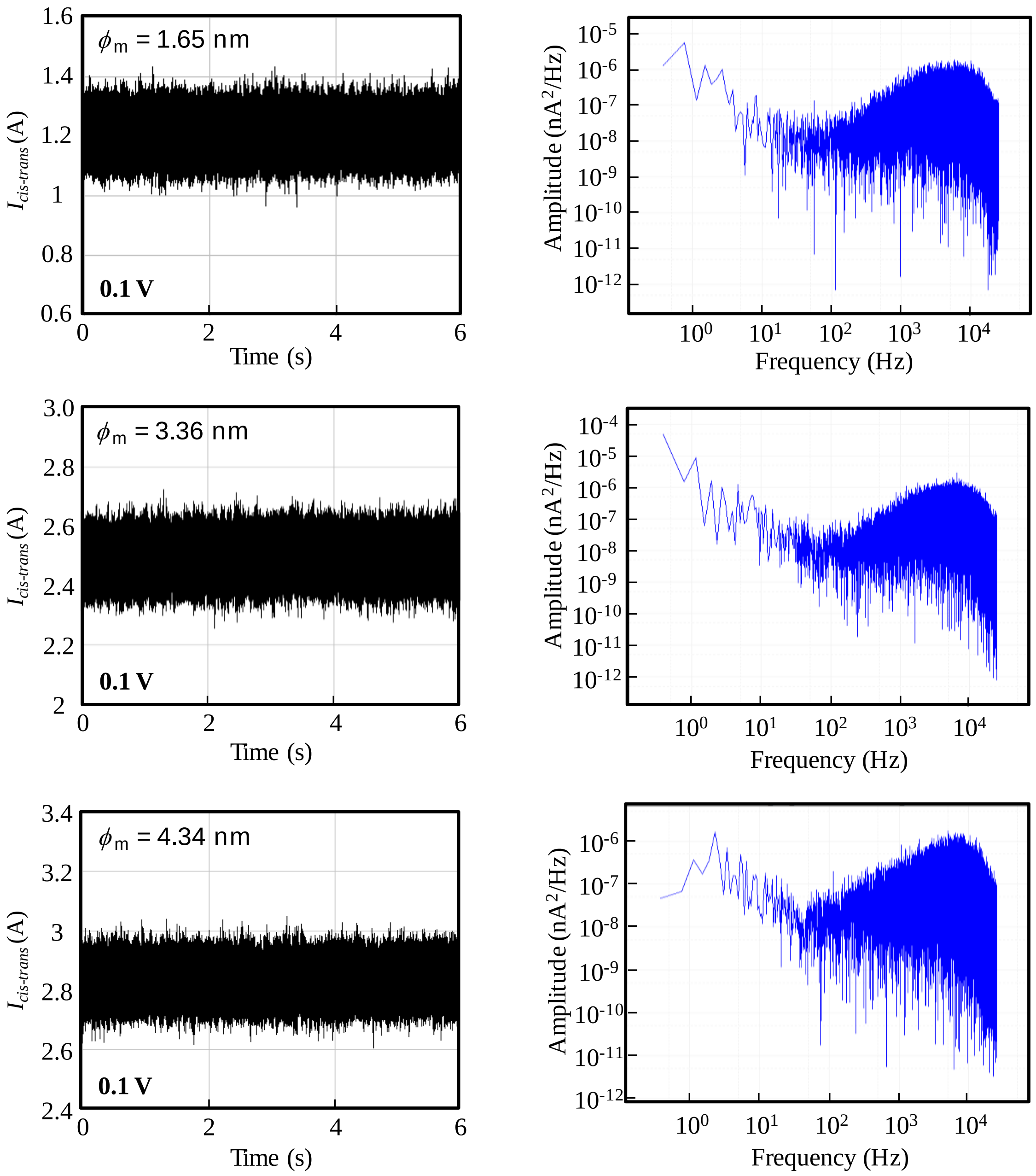

Fig. S-3

SI-4. TEM images of nanopores before and after ionic-current measurements

TEM images of 2 nanopores before and after long-term continuous measurement of the ionic current. The applied voltage was 0.1 V except for the initial short *I-V* measurements. The TEM images confirm that the nanopores widened after the ionic-current measurement.

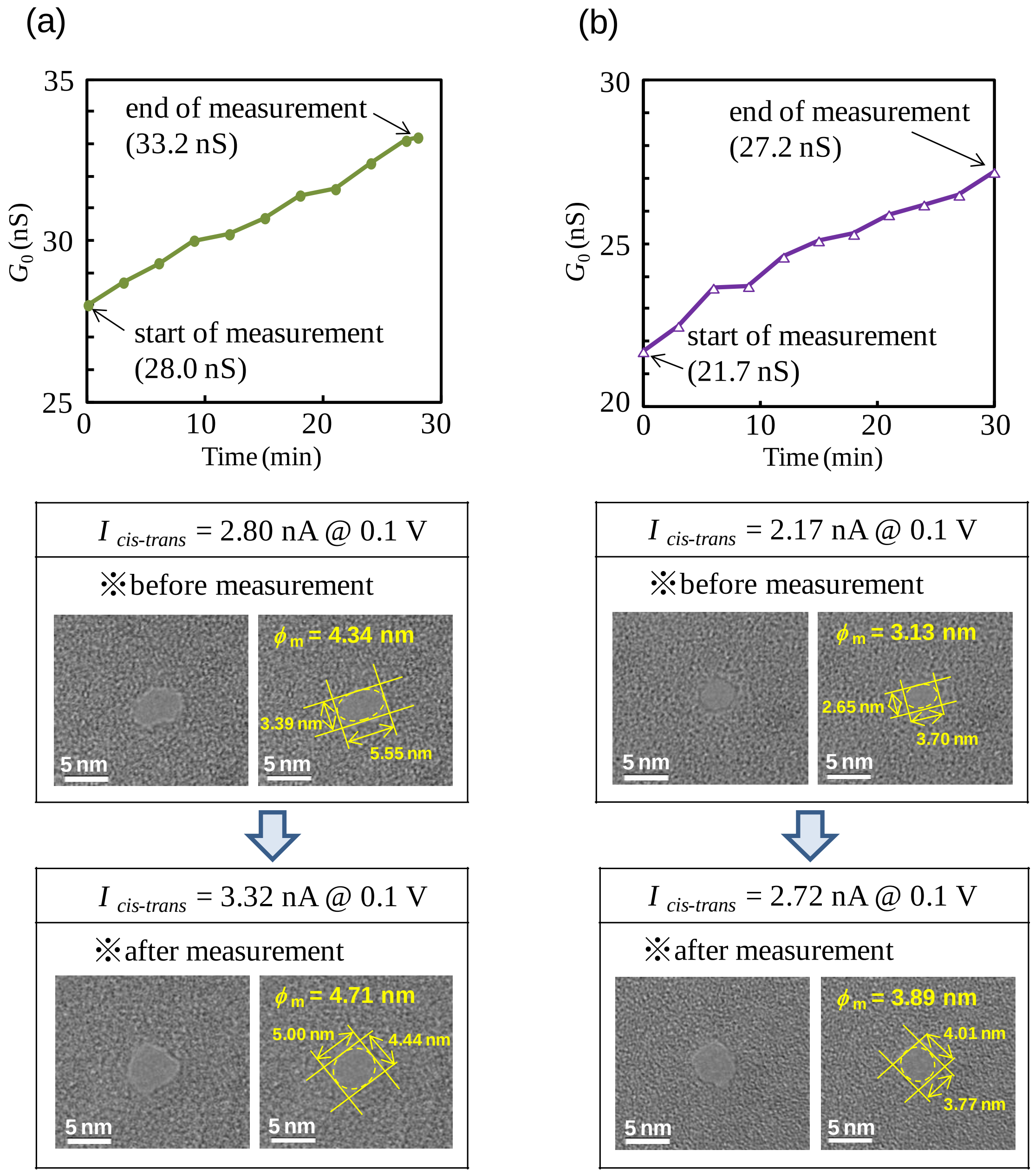

Fig. S-4

SI-5. Ionic-current blockades for several nanopores at 0.1 V

Ionic-current blockades (ΔI) measured from 4 different nanopores at 0.1 V are shown in Fig. S-5. ΔI_p shows variation (0.33 to 0.70 nA) between different nanopore chips. According to a report by Carlsen *et al.* [1], this variation may be attributed to variations in the amount of counterions surrounding the dsDNA.

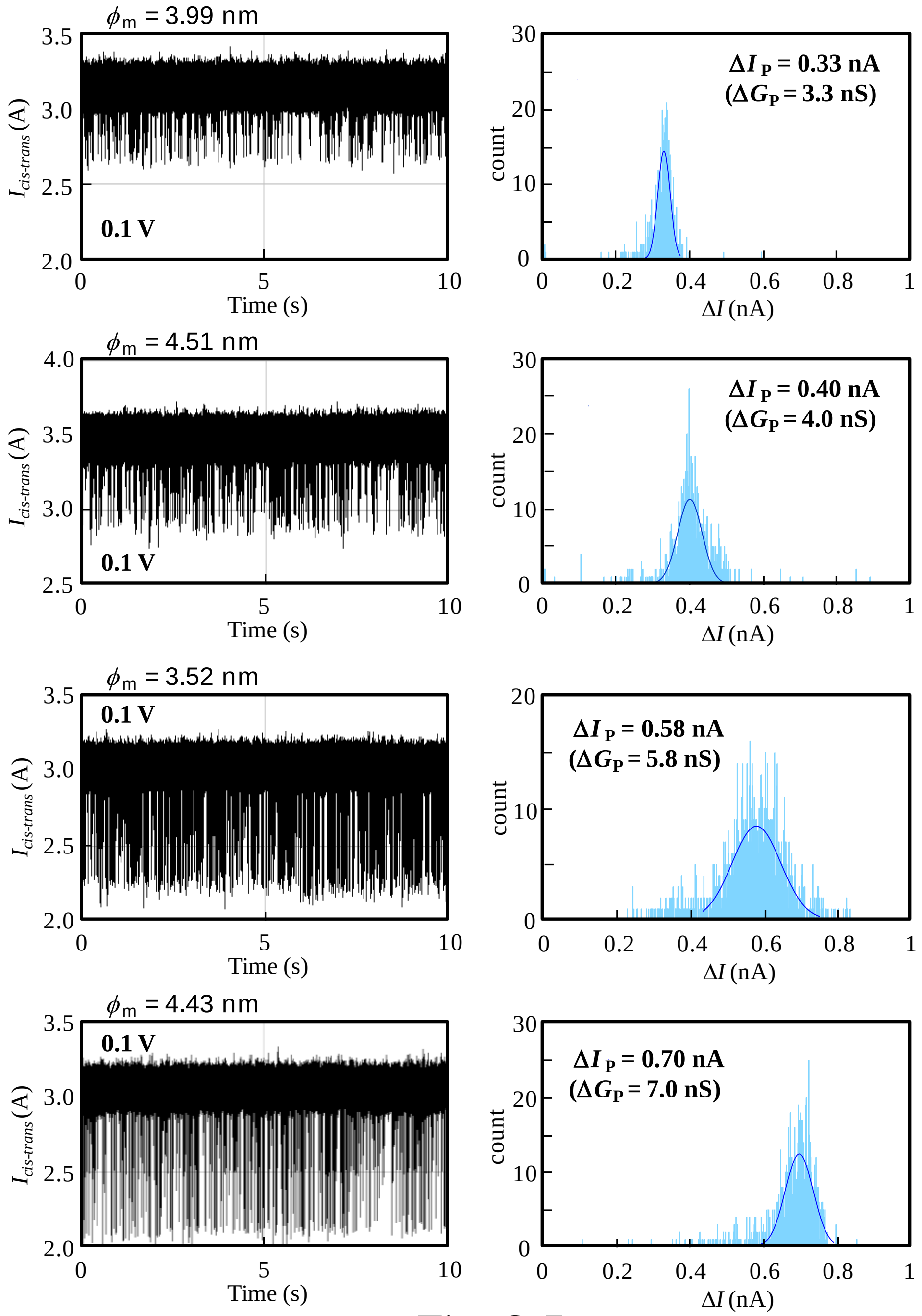

Fig. S-5

References

[1] Carlsen, A. T., Zahid, O. K., Ruzicka, J., Taylor, E. W., and Hall, A. R. Interpreting the Conductance Blockades of DNA Translocations through Solid-State Nanopores. *ACS Nano*, **8**, 4754–4760 (2014)
